# Management of Acute Stress Reactions in the Military: A Stepped Care Approach

**DOI:** 10.1007/s11920-022-01388-3

**Published:** 2022-12-20

**Authors:** Liana M. Matson, Amy B. Adler, Phillip J. Quartana, Connie L. Thomas, Emily G. Lowery-Gionta

**Affiliations:** Center for Military Psychiatry and Neuroscience, Walter Reed Army Institute of Research, 503 Robert Grant Avenue, Silver Spring, MD 20910 USA

**Keywords:** Acute traumatic stress, Intervention, Assessment, iCOVER, Medication, Pharmacologic

## Abstract

***Purpose of the Review*:**

This review highlights knowledge gaps surrounding the development and use of interventions for Acute Stress Reactions (ASRs). First, we propose that a stepped care approach to intervention for ASR be developed and utilized in military operational environments. A stepped care approach would include detection and assessment, followed by behavioral intervention, and then medication intervention for ASRs. Second, we discuss potential strategies that can be taken for the development of safe and effective ASR medications.

***Recent Findings*:**

ASRs commonly occur in operational environments, particularly in military populations. ASRs impact the safety and performance of individual service members and teams, but there are currently limited options for intervention.

***Summary*:**

Efforts to improve ASR detection and assessment, and development and delivery of ASR interventions for implementation in operational environments, will be critical to maintaining the safety and performance of service members.

## Introduction

Exposure to traumatic events is associated with a myriad of adaptive and maladaptive responses, encompassing both immediate and persistent behavioral, psychological, and somatic reactions. According to the Diagnostic and Statistical Manual for Mental Disorders–5th Edition (DSM-5), traumatic events are defined as exposure to actual or threatened death, serious injury, or sexual violence that is directly experienced, witnessed, or experienced by a close friend or family member or indirectly and repeatedly experienced as part of one’s occupation [1]. At the moment of the traumatic event, adaptive responses may minimize overwhelming stress symptoms and mitigate the risk for the development of longer-term post-traumatic symptoms [2, 3]. In contrast, maladaptive responses during trauma exposure may endanger safety and increase vulnerability to chronic post-traumatic symptoms.

**Fig. 1:**
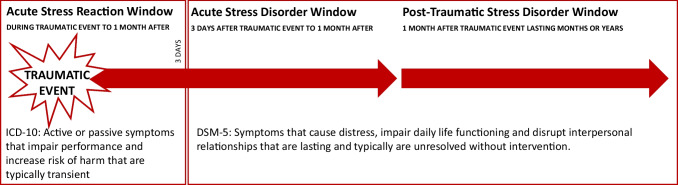
Timeline of post-traumatic event symptoms. Post-trauma symptoms can occur at any time following a traumatic event. Symptoms may resolve at any point, spontaneously or with treatment. The presence of symptoms at one point does not necessarily predict future symptoms. Theoretically, interventions to manage symptoms can be applied at any point during or following the traumatic event. Although an ASR can be present up to 30 days following trauma exposure, the first 72 hours are of critical importance for military operations, are the least well- understood or studied, and occur within a period that ASD or PTSD cannot be diagnosed

Acute stress disorder (ASD) is diagnosed when at least nine symptoms from categories including intrusion, negative mood, dissociation, avoidance, and arousal last from 3 days to 1 month following the traumatic event. Post-traumatic stress disorder (PTSD) is diagnosed if nine symptoms from the same categories persist for longer than 1 month [1]. If stress symptoms are experienced at the time of the traumatic event or within a month following the trauma, they may be classified as an Acute Stress Reaction (ASR) (Fig. 1). ASRs are described in the International Classification of Diseases (ICD) 11th Edition as a set of transient symptoms displayed in response to an “event or situation (either short- or long-lasting) of an extremely threatening or horrific nature” [4]. Symptoms may last hours to days following termination of the precipitating event and be emotional, somatic, cognitive, or behavioral in nature. Symptom presentation may be active (e.g., overactivity, anxiety) or passive (e.g., stupor, being in a daze, inactivity, depersonalization, derealization), though a comprehensive understanding of possible symptoms and their co-presentations is currently lacking.

Unlike PTSD and ASD, ASRs do not constitute a diagnosable condition and are generally believed to resolve without intervention. Accordingly, the 11th ICD edition now designates ASRs as normal reactions that scale to the severity of the precipitating event and the individual’s perception of it. Additionally, although an ASR can present up to 30 days following trauma, the classification also captures a period that is not captured by ASD or PTSD diagnoses. This period starts from the moment of traumatic stress exposure to 72 hours afterwards. ASRs can also cause significant distress and disrupt functioning, and their contribution to the onset of additional symptoms indicative of ASD or PTSD remains an open question.

ASRs have the potential to impact team-wide decisions and actions during and immediately following a traumatic event, especially for high-risk occupations like first responders and military service members. Recent self-reported data suggest that ASR-associated symptoms sufficient to impair soldier performance during traumatic events are common. Specifically, a study of soldiers who had been previously deployed and experienced combat revealed that as many as 17.2% experienced symptoms consistent with an ASR [5•]. Of these individuals, the majority reported that their performance was degraded for at least 5 minutes, and 19.2% reported that this disruption lasted more than 1 day. In a second retrospective study of pooled military samples, Adler, Svetlitzky, and Gutierrez (2020) found that nearly 50% of soldiers with combat deployment experience reported witnessing a team member experience an ASR-like episode [6•]. When asked about effects on performance, 39% of soldiers who witnessed the episode reported that the individual was unable to function and 31% reported that the individual was a risk to themselves or their unit. Symptoms witnessed included descriptions of the individual as emotionally overwhelmed (31%), confused (25%), erratic or agitated (19%), or detached (33%). Collectively, these data suggest that ASRs experienced in operational settings can have immediate impacts on performance, with potentially dire implications for the safety of service members and their unit. Considering the prevalence of ASRs in the military, it is important to develop interventions that sustain performance—possibly by reducing the duration and/or intensity of ASR symptoms. The period post-trauma and within 72 hours is of particular concern for the military, as affected service members are likely to still be experiencing the traumatic event or be in an operational setting during this acute period. In addition to the immediate benefit of managing ASRs, it is possible that early and effective intervention may reduce or prevent the development of chronic symptoms and disorders.

**Fig. 2:**
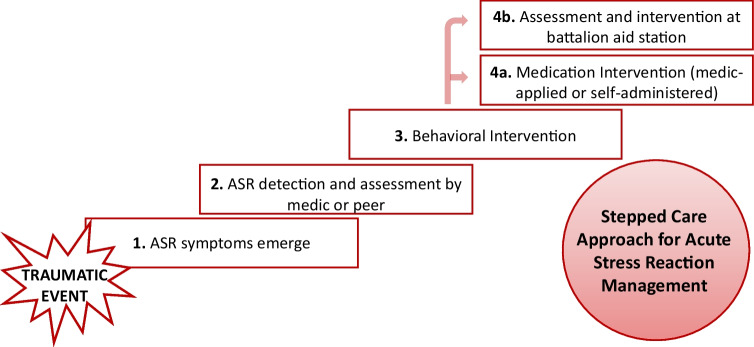
Stepped Care Approach to ASR symptom management. (1) ASR symptoms emerge. (2) ASR symptoms are detected and may be assessed by a peer or medic. (3) A behavioral intervention such as iCOVER is utilized. (4a) If symptoms do not resolve and an individual is unable to self-administer medication or a battalion aid station is not available, medication can be provided by a medic. (4b) If a battalion aid station is available and medic intervention is not urgently needed, step 4a may be skipped and assessment and intervention may occur at a battalion aid station. Throughout steps 2–4, monitoring by medic or provider for performance improvement or degradation should occur as much as feasible. If performance is still impaired in a previously treated individual, additional assessment and intervention may be applied at the battalion aid station. All steps may be repeated if symptoms re-emerge or, if available, the patient may need to be elevated to higher levels of care. As described in Fig. 1, symptoms may emerge immediately or up to 1 month following the traumatic event

Ideally, a toolkit of solutions would be available for safe and effective use to address the full range of ASR symptoms when there exists the potential for performance and safety to be impacted in the operational environment. One possibility is a stepped care approach in which ASR symptoms would be detected and the need for intervention assessed. If intervention is needed, a behavioral intervention would then be applied. If symptoms persisted, medication(s) to manage symptoms could be administered, taking into consideration the cost/benefit ratio of doing so given the extant operational situation (see Fig. 2). This model would offer the benefit of ASR symptom management while balancing the risk of applying medication-based interventions in operational settings, potentially even during the trauma itself. Overall, significant knowledge gaps exist that need to be studied in order to safeguard service members from the performance-impairing effects of ASRs and develop fast-acting, safe solutions that can be used in the near- and far-term, and especially in far-forward operating environments. In this review, we discuss some of the existing knowledge gaps and current and potential future states of a stepped care approach with a particular focus on ASR management by medications. Current Food and Drug Administration (FDA)-approved medications for PTSD take multiple weeks to reach treatment effects. Therefore, the identification of safe, effective, fast-acting medications is needed to address the acute symptoms associated with ASRs.

## The Stepped Care Approach to ASR Management in the Military Context

Ideally, a stepped care approach would be implemented by the military healthcare system to address the full range of ASR-induced performance deficits, with the initial point of care available during combat by peers or medics and follow on care available by providers at battalion aid stations. Because ASR symptoms sufficient to degrade performance may emerge during the trauma or in the days thereafter, a stepped care approach must be designed to be implemented as needed. Solutions should be implementable under extreme conditions, including during ongoing traumatic exposure and potentially during combat. Ideally, multiple intervention options will be available, enabling rapid escalation in the event that performance is not adequately restored by the initial intervention. It is also possible that behavioral and/or pharmaceutical interventions will need to be administered repeatedly to restore performance to acceptable levels or if symptoms resurface. As currently conceptualized, fellow unit members or medics could administer behavioral interventions while medics and/or providers at a battalion aid station would play a primary role in detecting, assessing, administering medications, and monitoring for ASR symptom resolution or persistence.

### ASR Detection, Assessment, and Monitoring

Figure 2 describes the role of detection, assessment, and monitoring over the course of a stepped care model for ASR intervention. Initially, an ASR might be detected and assessed by peers or medics to determine the need for an intervention. Monitoring thereafter can be used to determine the next step of care.

The current state of ASR detection in operational settings relies on fellow service members to recognize the behavioral symptoms of an ASR through subjective observation. This approach largely relies on the degree to which a first responder or peer is able to accurately observe behavioral changes. While subjective observation is useful, an objective, standardized approach developed to be more sensitive and rapid would allow the management of ASR symptoms perhaps even before they have fully manifested. To address this gap, objective tools to detect ASRs in operational environments are being actively developed. One emerging method for objectively detecting and monitoring an ASR could leverage passively collected sensor-based data [7]. The advantage of this approach is that artificial intelligence can be applied early in the onset of symptoms to detect risk for an ASR. One promising effort involves the passive collection of various features of speech. Vocal biomarkers have been shown to discriminate cases and controls for a number of clinical disorders including depression, but the predictive utility of these measures for ASRs in otherwise healthy individuals is currently unknown [8]. Changes in vocal biomarkers that co-vary with ASR symptoms could serve as a meaningful non-invasive detection tool in operational environments. Overall, the use of biosensors that detect physiological changes associated with ASRs could prompt peers, leaders, and healthcare providers to intervene early and prior to more sustained or severe symptom manifestation.

Unfortunately, data on the assessment of acute stress experienced during a combat-relevant time window are limited. A review of the relevant studies assessing peri-traumatic distress symptoms, revealed that most relied on self-report measures such as the Impact of Events Scale (IES) and PTSD Checklist (PCL) that were administered within days of the traumatic event [9, 10]. These measures appear to be useful in assessing risk, but by definition, they rely on an individual’s ability to accurately recall and respond to questions. However, if an individual is in an acute dissociative or panic-like state, it may be extremely difficult to complete a valid assessment. The Clinician-Administered Dissociative States Scale is used in some military contexts, though narrow evaluations of dissociation alone do not capture the full scope of ASR symptoms. Therefore, it is critical to specify criteria that enable medics or other providers or team members to rapidly assess the severity of an ASR. In recognition of this, work has begun to develop modifications to medic training that would enhance their ability to detect and assess ASR, and thereby facilitate on-the-spot decision-making, as medics are often the first providers that would encounter an individual experiencing an ASR. New efforts are also underway to develop an assessment approach for medics to use, as current medic training is not comprehensive enough to tackle the challenges of formally evaluating ASRs. Although a behavioral intervention may be administered by individual teammates without a need for formal assessment, medication administration will likely require prior medic or other provider assessment.

In addition to detection and assessment, ongoing monitoring for the efficacy of the behavioral or medication intervention to resolve ASRs should be used. This is particularly important for medications that could have performance-impacting side effects. Monitoring for symptom improvement will help determine if there is a need for further intervention. For example, symptoms may re-emerge after initial dosing, or impairments may persist and require a higher level of care. As described in Fig. 2, monitoring can occur at multiple points in the stepped care model and be performed by medics and/or providers at battalion aid stations. While it is possible that the same detection or assessment tools could be used for monitoring, it is also possible that there is a need to develop specialized tools for monitoring.

### Interventions for ASRs

There is broad agreement that mental health support is necessary in operational settings. However, providing mental health support during operations—arguably where and when it may be most effective—is challenging. Current practice relies on area support from providers assigned to combat brigades and/or Combat and Operational Stress Control units [11•, 12]. This model has been used with success to stabilize patients as close to operational settings as possible and return them to duty, or to medically evacuate them to higher levels of care depending on symptom severity and persistence. However, future combat is expected to be large-scale and fought across multiple domains including air, land, space, cyber, and electromagnetic domains [13]. In preparing for these types of multi-domain operations, it is anticipated that the USA will not have ready access to secure communication capabilities and air support for medical evacuations. Further, ready access to behavioral health providers, even via remote telehealth communications, is expected to be limited. In light of these anticipated challenges, the US Army has invested in the delivery of care as close to the frontlines as possible. This operating concept places fellow service members and medics at the center of care provision, and it is anticipated that they will be responsible for stabilizing and returning to duty to those who experience ASRs. Moreover, there will be a heightened need to ensure that individuals experiencing psychiatric symptoms resume functioning as quickly as possible in order to return to duty and ultimately, restore combat power. With this concept of future warfare in mind, behavioral interventions and medications tailored for use in operational settings are now under development. Depending on the level of care needed and the type of care available, these solutions are designed to be applied during operations at the front lines or at battalion aid stations. If a service member does not respond to initial interventions in a forward-deployed environment, they may require evacuation to a higher echelon of medical care with more robust treatment options and monitoring. Because patient movement may be delayed due to the operational environment, medics or providers at the battalion aid station require education on triage, monitoring, and treatment.

#### Behavioral Intervention

For the past several decades, pre-deployment training designed to promote resilience and mental health have been provided to deploying units, though these trainings have not specifically addressed the management of ASRs. Psychological first aid is currently the first-line treatment for acute traumatic stress effects. This treatment is limited because it was developed to address civilian traumatic stress and does not address the return to performance that would be needed in operational settings [14]. Therefore, this gap has resulted in service members managing ASR symptoms by responding instinctually or making the best guess about what might work [15•]. Across two independent samples, the most common strategies service members reported included calmly speaking to or yelling at the individual experiencing the ASR, as well as directing them to perform a simple task [6•]. Though less frequently reported, some reported shaking, hitting, or pushing the individual in an attempt to resolve the ASR, and others expressed that they did not know how to respond. These responses indicate the need for both structured training and interventions to enable all unit members to address ASR symptoms effectively at the moment, even in the midst of combat.

A recently developed intervention was specifically designed to meet this need, with the goal of restoring performance as quickly as possible. This intervention is based on one originally created by the Israel Defense Forces and consists of a series of steps service members can use with team members experiencing an ASR at the frontlines [16]. The US version of this training, iCOVER, also includes steps designed to refocus the affected individual through connecting, offering commitment, engaging the brain’s automatic processes through simple questions, grounding the individual by providing a brief sequencing of events, and requesting purposeful action. Notably, iCOVER can be used by anyone following a single session of training and is designed to address both active and passive ASR symptoms. Initial studies of iCOVER training have demonstrated its feasibility, acceptance, and positive impact on attitudes, as well as an increase in the ability of individuals to enact these steps in realistic training scenarios [15•]. More recently, over 90% of National Guardsmen undergoing iCOVER training in preparation for deployment found the training important, relevant, and useful [5•]. Importantly, iCOVER training increased service members’ own confidence to help a fellow unit member manage an ASR and also increased confidence in their fellow unit members to help them if they experienced an ASR. Currently, iCOVER is being disseminated in the US Army as part of Deployment Cycle Resilience Training and has been adapted by militaries in other nations as well. Overall, though additional efficacy testing is needed, iCOVER is a major advance in the management of ASRs in an operational setting.

#### Medication Intervention

In the event that ASR symptoms persist after behavioral intervention and symptoms continue to be severe enough to impact performance or put the service member or team members in danger, medications could be used to alleviate symptoms and restore performance. These medications may be self-administered or administered by a medic on the frontlines or a provider at a battalion aid station, depending on the capability of the service member requiring treatment and proximity to the battalion aid station. As no medications designed for this context are currently available, the need for rapid development is critical.

Although the neuropathophysiology between ASRs and ASD/PTSD may differ, it would be reasonable to postulate that medications used for ASD and PTSD may be useful for managing ASRs. Currently, there are two FDA-indicated medications for PTSD, sertraline and paroxetine. Both medications are selective serotonin reuptake inhibitors (SSRIs) and are also used to treat ASD off-label. However, neither is particularly promising for the treatment of ASRs because symptom relief is not immediate upon initiating SSRI treatment, with symptom relief often taking weeks to sometimes months [17]. Another reason is that even once this period has elapsed, many patients find limited therapeutic benefit of SSRIs for trauma-related symptoms [18]. Off-label prescribing of medications to treat long-lasting post-trauma symptoms is common, but SSRIs are not an ideal treatment for ASD because they take weeks to reach therapeutic effect and may be stopped prematurely due to lack of benefit. Other SSRIs, monoamine oxidase inhibitors (MOA-Is), serotonin-norepinephrine reuptake inhibitors (SNRIs), benzodiazepines, and atypical antipsychotics are most commonly tried [19]. Although off-label use of these medications for the management of ASD/ PTSD may be supported somewhat by research and clinical practice, none of these medications have been evaluated for the management of ASRs. In addition, the side effect profiles of these medications may potentially be problematic in operational settings, particularly for MAOIs and antipsychotics.

Rather than searching among existing treatments for long-term trauma-associated symptoms, we propose that new medication identification efforts be initiated that focus on treatment efficacy for specific ASR symptoms. Because ASR symptoms can present at both ends of the active versus passive spectrum, it is likely that two or more medications may be needed to adequately restore performance, depending on symptom presentation. For example, medications addressing active symptoms may reduce sympathetic activity or amygdalar drive, while medications addressing passive symptoms may activate these mechanisms. In addition to demonstrating efficacy in restoring performance and basic safety and tolerability requirements, several other conditions must be met for medications to be suitable for operational use. For example, medication development efforts must account for the specific needs of service members operating in austere, often hostile, environments with extreme weather conditions and limited storage options. As such, potential solutions must be carefully vetted for shelf stability, ease of administration, risk of abuse/dependence, and side effects that may interfere with operational performance and other undesired effects (e.g., interactions with common medications or foods, diuretic or dehydrating effects). Where and by whom medications will be administered must also be considered (e.g., during operations at frontlines versus at a battalion aid station, and self-administered versus assisted administration, respectively). Additionally, as medics may need to administer medications for ASRs, it is important to consider which medications should be included in a space-limited medic bag or at a battalion aid station.

## Approach to Developing Medications to Manage ASRs

In light of the unique challenges of developing medications for use by service members in operational settings combined with the long lead times and high failure rates traditionally encountered when developing medications to address psychiatric symptoms, a well-defined approach to medication development for ASR management is essential. Acceptable versus optimal metrics for key criteria are also needed. At a minimum, fieldable medications must be (1) safe and well-tolerated for use during operations, (2) effective in at least partially improving ASR-induced performance degradation, (3) easy to administer even to individuals who are incapacitated or otherwise unable to self-administer medication, and (4) rapid-acting. Optimal solutions should meet these acceptability standards and also aim to (1) have no or few limitations on use during operations, (2) fully restore ASR-induced performance degradation, and (3) reduce the risk of developing chronic psychiatric symptoms. To more rapidly deliver solutions for ASR management without compromising the development of medications that are tailored to this purpose, a dual-pronged development approach is appropriate. One effort focuses on the rapid delivery of acceptable solutions for use in the near term. The other focuses on the delivery of optimal solutions for use in future conflicts.

### Development of Medications for Operational Use in the Near Term (5–7 Years)

**Fig. 3:**
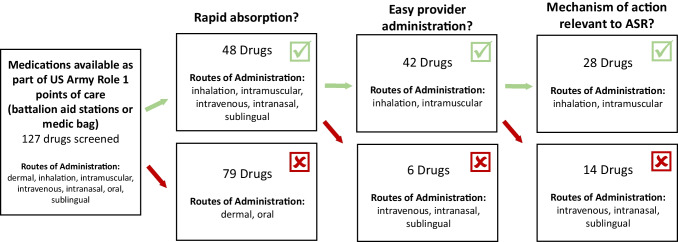
Downselection process for testing medications to be applied to individuals who are unable to self-administer. Downselection process for medications currently available in US Army battalion aid stations or medic bags to test for performance effects and ASR symptom management efficacy. Downselection criteria were fast absorption rate, ease of administration, and mechanism of action. Of 127 medications available, 28 medications were found to be worthy of consideration for performance testing. Preferred criteria were administration routes that are (1) rapid and (2) relatively easy for providers to administer and (3) mechanisms of action previously implicated in the stress response

**Fig. 4:**
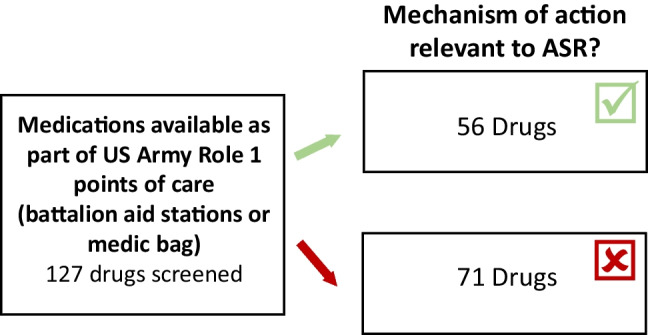
Downselection process for testing medications to be applied to individuals who are able to self-administer. Downselection process for medications currently available in US Army battalion aid stations or medic bags to test for performance effects and ASR symptom management efficacy. Downselection criteria were fast absorption rate, ease of administration, and mechanism of action. Of 127 medications available, 56 medications were found to be worthy of consideration for performance testing. Preferred criteria was a mechanism of action previously implicated in the stress response

Given the need to develop a workable and streamlined strategy for identifying medications to apply in the near term, we believe it is useful to consider what is already available to medics or providers in the operational environment, specifically those medications that are already included in medic bags or at battalion aid stations. Though these medications are used for other indications, it is possible that some of them could be repurposed for off-label management of ASR symptoms. Because they are already in use in operational settings, their safety, tolerability, and other usability requirements have already been evaluated. Conceivably, medications could be administered in one of two scenarios: (1) to an individual in an acute panic-like or dissociative state who is unable to self-administer a medication or (2) to an individual that is experiencing ASR symptoms but is able to self-administer under the care of a medic or provider. For the first scenario, criteria such as ease of administration, rapid action, and mechanism of action can be used to down-select candidate medications for efficacy testing (Fig. 3). A review applying these criteria to 127 medications currently available as part of the US Army Role 1 assemblages (either part of medic bags or at available at battalion aid stations) resulted in the identification of 28 potential candidate medications for further testing for use in individuals unable to self-administer medication [20]. For the second scenario, the mechanism of action was the criterion applied (Fig. 4). This down-selection resulted in the identification of 56 potential candidate medications for further testing for use in individuals who are able to self-administer medication. For both examples, the most promising candidates were further down-selected based on demonstrated efficacy of their mechanisms of action (i.e., whether they affect processes implicated in the etiology of traumatic stress effects) and/or because there is evidence of efficacy for preventing or treating PTSD.

We propose a two-step sequential testing process to determine the suitability of each candidate medication for ASR management in operational settings. This two-step process will help to delineate the use parameters, determine their efficacy for treating active and/or passive symptoms, and build a body of evidence that supports changes to relevant military training and practice guidelines. Both steps would require blinded, placebo-controlled clinical trials conducted in populations with characteristics relevant to service members. In the first step, candidate medications would be tested for effects on military-relevant performance metrics. To increase the relevance of this testing for managing ASRs, performance should be tested under stressful conditions whereby study participants are sympathetically activated. Candidate medications found to have positive or null effects on performance, suggesting that they improve or sustain performance, would be considered for further assessment in step two.

In step two, medications down-selected from the first step would be tested for effects on ASR symptoms and simple performance measures. Because of the difficulty of capturing ASRs in operational settings, this study would be best undertaken in a proxy population and setting, such as in individuals admitted to hospital emergency departments following an acute traumatic event. This study would follow a similar design and methodology as those used in the Advancing Understanding of Recovery After Trauma (AURORA) study [21•]. In that multi-site study, participants were recruited from emergency departments and enrolled within 72 hours of experiencing a traumatic event that caused distress but not serious physical injury. Assessments were collected in the emergency department and at 2 and 8 weeks post-event. In a similar manner, step two testing would include the administration of medication at enrollment and follow-up assessments of ASR symptoms and performance conducted during the days and weeks following the traumatic event. Treatment efficacy for both passive and active symptoms would be assessed. PTSD symptoms would also be assessed at later points to test the hypothesis that acute treatment of ASR symptoms reduces the risk of future PTSD symptoms. Medications that reduce ASR symptoms and improve performance would be considered for advanced development.

A two-step testing process could also be used to assess additional medications that are approved for other indications by the Food and Drug Administration (FDA) but are not currently available in operational settings, although additional assessments for operational use and fielding would likely be needed.

### Development of Medications for Operational Use in the Far Term (15–20 Years)

In parallel to the development of medications for the near term, basic science efforts to develop novel medications that meet optimal criteria are ongoing. Basic preclinical science studies to identify the pathophysiological processes that underlie ASRs can potentially identify targets of opportunity for the development of ASR-specific medications. Capturing ASRs in people as they are occurring can be costly in terms of both resources and time, thereby complicating the process of collecting biosamples to cultivate new medication targets. Likewise, clinical trials of candidate medications to establish efficacy are expensive. Preclinical investigations are cost- and time-effective for providing these insights through the use of virtual, synthetic, cellular, and animal models, with the ability for medications to be evaluated in parallel or sequentially over a relatively short period of time.

Animal models have been integral to the understanding of the effects of a variety of stress exposures on neurophysiological and neurobehavioral processes [22]. Many models rely on one form of stress presented in a single exposure or over repeated exposures to provoke measurable responses, typically behavioral, endocrine, or physiological in nature. To best model traumatic events, animal models must have ecological validity for inducing perceptions of a direct threat to life. For example, our group has developed a single-day stress procedure called Military-Relevant Complex Traumatic Stress (MRCTS) to model a military-relevant traumatic event by incorporating the perception of a direct threat to life with additional elements of traumatic events that service members may encounter in operational settings [23]. As part of far-term development, medications that are found to restore behavioral performance, improve surrogate endpoints after traumatic stress exposure, and that have favorable pharmacological characteristics can be transitioned to advanced testing and development. Throughout this process, emerging technologies can be applied to enhance down-selection decisions and speed progress. The ultimate objective is to deliver novel FDA-approved medications that fully restore performance, rapidly alleviate ASR symptoms, and reduce the risk of PTSD symptom onset so that service members can remain safe and maintain performance during high-stress operations.

## Future Directions and Conclusions

Many knowledge gaps remain in the areas of ASR detection, assessment, and intervention (see Table 1). Chief among them is a comprehensive understanding of the full range of ASR symptoms. Historically, studies of acute trauma have typically relied on self-reports collected weeks to years after the traumatic event, and many of these have failed to focus specifically on ASR assessment or intervention. In fact, for many of these studies, the focus has been on specific psychiatric symptoms, especially peritraumatic disassociation and distress. Although these efforts are important and useful, a more comprehensive understanding of the psychiatric, somatic, cognitive, and motor symptoms that impair performance is needed to inform the development of ASR-targeted medications. It will also be important to characterize the pattern of symptoms to determine whether there are ASR subtypes, and if so, to determine the population penetrance of each—especially if these subtypes are differentially responsive to the various interventions, in which case it will be important to prioritize development of subtype-specific interventions accordingly.

**Table 1:** Key questions in the field of Acute Stress Reaction (ASR) management

Question
What is the full range of ASR symptoms?
What are the most common ASR symptoms? What is the prevalence of ASR symptoms?
Do ASR symptoms co-vary?
What are the most common ASR phenotypes?
What is the relationship between ASR symptoms/ phenotypes and chronic symptom development?
What is the relationship between ASR symptoms/ phenotypes and the risk of ASD/ PTSD onset?
What are the neurophysiological underpinnings of ASR symptoms? Do they differ for active versus passive symptoms? Are different medications needed to address active versus passive symptoms?
What are the predictors of ASR symptoms?
Do men and women differ in their ASR symptom presentations? Do the neurophysiological underpinnings of ASR symptoms differ between men and women?
How do we effectively assess the severity of ASRs? Is there a threshold for medication administration? Can we use the same standards to monitor medication efficacy or lack thereof?
If an ASR occurs in parallel with trauma, is intervention beneficial or detrimental for long-term responses?
If ASR symptoms re-occur will additional dosing with initial medication attenuate symptoms? What symptoms may be treatment resistant?

Relatedly, the lack of knowledge of the extent to which specific ASR symptoms reflect activity, reactivity, or lack of activity in specific neural circuits leaves key questions unanswered. Do passive and active symptoms involve distinct neural pathways or are the same pathways differentially recruited? Will different medications be needed to target specific ASR symptoms and/or subtypes? Another key gap in knowledge is related to the time course of ASR symptom presentation. Given that our stepped care approach is based upon the emergence of ASR symptoms rather than time elapsed from the traumatic event, it is reasonable to hypothesize that the behavioral and pharmaceutical interventions discussed could be used whenever ASR symptoms emerge rather than time-locked to the event itself. However, the efficacy of medications might vary as a function of time since trauma exposure, especially because the engagement of underlying physiological processes probably changes over time. Similarly, the frequency at which putatively resolved ASR symptoms re-emerge at levels sufficient to impair performance requires further investigation. If re-emerging symptoms are found to be common, medication development efforts will need to determine the safety and efficacy parameters for repeated dosing of one medication, or sequential dosing of two or more medications to fully manage symptoms and restore operational performance. Finally, the relationship between ASRs, possible ASR subtypes, and the likelihood of chronic psychiatric symptoms must be assessed since this information will define the scope of medication development and may provide evidence supporting early treatment approaches to prevent ASD/PTSD.

Historically, the development of tools and interventions to support ASR management in operational settings has been largely overlooked. It is also difficult to study ASR during the co-occurrence of trauma since traumatic stress exposures are largely unpredictable. Therefore, most studies have had to rely on retrospective self-report to understand the presentation of an ASR. Efforts to improve ASR detection, assessment, development, and delivery of ASR behavioral interventions and medications are underway and would benefit from a greater overall understanding of ASR symptoms and their underlying neurophysiology, effects on performance, prevalence, and contribution to the onset of chronic psychiatric symptoms. Medication development efforts must be attuned to external factors that influence the safe and effective use of medications to manage ASRs in operational settings. These efforts must be structured to deliver solutions as rapidly as possible, with a clearly defined criterion for near-term and long-term implementation. In the near-term, practice guidelines can be pursued for the use of previously fielded medications for off-label use in operational settings by the Department of Defense to include ASR management and providing training on the conditions and guidelines for use. In the far-term, optimized solutions can be developed to safely and effectively treat the spectrum of ASR symptoms, with minimal effects on performance. These efforts must be responsive to the current and future needs of service members as the nature of warfare is expected to shift to relying on small teams in isolated areas that do not have the kind of medical support more traditionally expected. Though the demands of this environment will require investment in terms of time and resources, the option of effective ASR management through medications will provide a backstop for potentially unmanageable symptoms that degrade operational performance and put service members and their units in danger. Ultimately, the refinement of a stepped care approach to ASR management in operational settings will protect service members by either restoring them to duty or determining when they are combat ineffective.

## Declarations

### Disclaimer

This material has been reviewed by the Walter Reed Army Institute of Research, and there is no objection to its presentation and/or publication. The opinions or assertions contained herein are the private views of the authors and are not to be construed as official or as reflecting the position of the Department of the Army or the Department of Defense.
